# Central masked adjudication of stroke diagnosis at trial entry offered no advantage over diagnosis by local clinicians: Secondary analysis and simulation

**DOI:** 10.1016/j.conctc.2018.11.002

**Published:** 2018-11-10

**Authors:** Peter J. Godolphin, Trish Hepburn, Nikola Sprigg, Liz Walker, Eivind Berge, Ronan Collins, John Gommans, George Ntaios, Stuart Pocock, Kameshwar Prasad, Joanna M. Wardlaw, Philip M. Bath, Alan A. Montgomery

**Affiliations:** a Stroke Trials Unit, Division of Clinical Neuroscience, University of Nottingham, Nottingham, UK; b Nottingham Clinical Trials Unit, University of Nottingham, Nottingham, UK; c Department of Internal Medicine, Oslo University Hospital, Oslo, Norway; d Tallaght Hospital, Tallaght, Ireland; e Hawke's Bay District Health Board, Hastings, New Zealand; f Department of Medicine, University of Thessaly, Larissa, Greece; g Department of Medical Statistics, London School of Hygiene and Tropical Medicine, London, UK; h All India Institute of Medical Sciences, New Delhi, India; i Neuroimaging Sciences, Centre for Clinical Brain Sciences, University of Edinburgh, Edinburgh, UK

**Keywords:** Adjudication, Diagnosis, Clinical trial, Stroke

## Abstract

**Background:**

Central adjudication of stroke type is commonly implemented in large multicentre clinical trials. We investigated the effect of central adjudication of diagnosis of stroke type at trial entry in the Efficacy of Nitric Oxide in Stroke (ENOS) trial.

**Methods:**

ENOS recruited patients with acute ischaemic or haemorrhagic stroke, and diagnostic adjudication was carried out using cranial scans. For this study, diagnoses made by local site clinicians were compared with those by central, masked adjudicators using kappa statistics. The trial primary analysis and subgroup analysis by stroke type were re-analysed using stroke diagnosis made by local clinicians, and simulations were used to assess the impact of increased non-differential misclassification and subgroup effects.

**Results:**

Agreement on stroke type (Ischaemic, Intracerebral Haemorrhage, Unknown stroke type, No-stroke) was high (κ = 0.92). Adjudication of stroke type had no impact on the primary outcome or subgroup analysis by stroke type. With misclassification increased to 10 times the level observed in ENOS and a simulated subgroup effect present, adjudication would have affected trial conclusions.

**Conclusions:**

Stroke type at trial entry was diagnosed accurately by local clinicians in ENOS. Adjudication of stroke type by central adjudicators had no measurable effect on trial conclusions. Diagnostic adjudication may be important if diagnosis is complex and a treatment-diagnosis interaction is expected.

## 1 Introduction

Clinical trials in acute stroke often recruit many thousands of participants making them complex, lengthy, and expensive. In many stroke trials, key endpoints, adverse events, or diagnoses qualifying for trial entry are adjudicated by independent experts. Independent, central adjudication may be conducted by one individual or a panel of experts, who may work independently or convene as a committee, with agreed procedures for assigning definitive values, usually blinded to treatment allocation whenever possible [1]. The adjudication procedure is believed to protect against bias resulting from differential misclassification [2,3], and to improve precision of treatment estimates by reducing ‘noise’ from random errors. This is especially important in trials where events are rare, in which a small degree of misclassification can have a large impact on study findings [2,3] or where the event is subjective such as some clinical diagnoses. Adjudication also introduces a level of quality control to detect poorly trained or performing investigators.

Central adjudication is commonly included in cardiovascular studies [4,5], with conflicting evidence as to the value of adjudication of endpoints [[6], [7], [8], [9], [10], [11], [12], [13]] compared with simply using endpoints assigned by local clinicians or investigators at participating research sites. There is little research evidence regarding the importance of diagnostic adjudication, where diagnosis is not used as an endpoint, but is used to diagnose patients at trial entry. Diagnoses made at trial entry can be used to define eligibility, as a stratification or minimisation factor, as a covariate in a regression model, or to specify categories in a subgroup analysis.

Stroke is a clinical diagnosis that can be further subclassified based on the results of further investigations, including brain and vessel imaging and cardiac examinations. Given the complex nature of stroke subtypes [14], stroke diagnoses are commonly adjudicated by independent experts in clinical trials. Ninomiya et al. [11] found that adjudication of stroke type and cause of death as study endpoints had no substantive impact on treatment effect estimates in their trial. However, stroke diagnosis was an endpoint, rather than a criterion for inclusion. While adjudication of endpoints has the greatest potential to influence trial results and therefore has received greatest attention as to its value, misclassification of entry criteria might also introduce bias, affect the precision of effect estimates or reduce statistical power [15]. However, we are not aware of any such investigation of the value of central adjudication of the diagnosis qualifying for trial inclusion.

The aim of this study was to investigate the value of central adjudication of stroke type at trial entry in a secondary analysis of a large acute stroke trial. The three objectives were to [1]: compare stroke diagnoses made by local clinicians and central masked adjudicators [2]; assess the impact of adjudication on the primary analysis and the subgroup analysis by stroke type [3]; using simulation, explore the effects of increasing levels of misclassification of diagnosis and introducing a subgroup effect by stroke type on analyses.

## 2 Materials and methods

### 2.1 Efficacy of Nitric Oxide in stroke (ENOS) trial

The Efficacy of Nitric Oxide in Stroke (ENOS) trial examined the safety and efficacy of glyceryl trinitrate (GTN) versus no GTN in patients with acute ischaemic or haemorrhagic stroke. Independent expert assessors, referred to in this paper as adjudicators, who were masked to treatment allocation, centrally assessed CT and MRI scans to inform diagnosis of stroke type. The primary outcome was functional outcome after stroke, measured using the modified Rankin Scale (mRS) at day 90 by outcome assessors who were masked to treatment allocation. The trial recruited 4011 patients from 173 sites, across 23 countries on five continents. The primary outcome was analysed using ordinal logistic regression, and the adjusted common odds ratio (OR) for worse outcome with GTN versus no GTN was 1·01 (95% CI 0·91 to 1·13; p = 0·83). The protocol, statistical analysis plan, and main results for ENOS have been described in detail elsewhere [[16], [17], [18]].

### 2.2 Diagnosis of stroke type

After enrolment into the ENOS trial, all participants had a CT (or MRI) scan at baseline or within seven days (referred to as baseline scan), and if possible again after seven days (referred to as follow-up scan) to assess evolution of the stroke lesion. Each scan was analysed by local clinicians, who then used information from the baseline scan, follow-up scan if available, input from the local radiology team, and clinical history and assessment of the participant between admission and discharge, in order to assign a clinical diagnosis for each participant (referred to as Local clinician diagnosis). The following diagnoses were made: Ischaemic stroke, intracerebral haemorrhage, unknown stroke and no stroke. All scans were then sent electronically to the central trial team.

A team of independent, central adjudicators, masked to treatment allocation and Local clinician diagnosis, assessed all brain scans. They recorded their assessment using a specially designed questionnaire that captured information on the presence of stroke, haemorrhage, occluded arteries, Alberta stroke program early CT score [19], mass effect, white matter disease, atrophy, and other visible lesions. This information was used to determine an adjudicator diagnosis of stroke type for both baseline and follow-up scans. A final diagnosis of stroke type for each participant (referred to as Central adjudication diagnosis) was assigned using an algorithm that assessed whether diagnoses from local clinicians and adjudicators sufficiently agreed, otherwise stroke diagnosis was allocated on a case-by-case basis.

Central adjudication diagnosis was assigned using all available information from both local clinicians and adjudicators, and was thus considered in this study as the ‘gold standard’. Local clinician diagnosis represents the diagnosis of stroke type in ENOS if no central adjudication had taken place. In the ENOS analyses, stroke type at trial entry was included in between-group comparisons as a baseline covariate, and as a subgroup variable to investigate any differential effects of the interventions according to stroke type. The main ENOS analyses used Central adjudication diagnosis of stroke. The analyses presented here compared the main ENOS analyses with analyses conducted using Local clinician diagnosis of stroke, thus allowing an investigation into the value of adjudication of a baseline variable in ENOS.

### 2.3 Simulated misclassification of stroke type and simulated subgroup effect

Statistical simulations were created to [1]: increase the extent of misclassification of Local clinician diagnosis of stroke compared with the gold standard Central adjudication diagnosis [2]; introduce an interaction (subgroup effect) between ENOS treatment arm and stroke type. These simulations enabled us to investigate the effects of misclassification on the ENOS primary analysis and on subgroup analysis, for both the subgroup effect observed in ENOS and for a subgroup effect introduced by simulation. The magnitude of the treatment-stroke type interaction was increased in simulation as there was no statistical evidence of a subgroup effect in the observed ENOS dataset.

In simulated datasets, the misclassification of Local clinician diagnosis observed in ENOS was increased by factors of 3, 5, 10, 15 and 20 (referred to as SX3, SX5, SX10, SX15 and SX20 respectively). We also introduced a subgroup effect by reducing mRS score by 1 point for 10% of participants with an Ischaemic stroke, and increasing mRS score by 1 point for 30% of participants with an Intracerebral Haemorrhage, with mRS scores for all participants constrained to be in the normal range 0–6. All participants with an altered mRS score were in the GTN arm of the trial. For more detailed simulation methods, please consult Supplementary File S1.

### 2.4 Statistical methods

Categorical variables were described using N (%). Observed agreement between Local clinician and Central adjudication diagnoses was quantified using unweighted kappa statistics.

Using observed ENOS data, the effect of GTN treatment on mRS score was estimated as in the ENOS trial main report, using ordinal logistic regression models, adjusted for stratification and minimisation variables. Models including Local clinician and Central adjudication diagnosis of stroke type as a covariate were fitted separately and the estimated effects of GTN treatment from the two models were compared using a test of homogeneity. Similarly, subgroup effects were estimated by fitting an interaction term between GTN treatment and stroke type according to either Local clinician or Central adjudication diagnosis.

The primary trial analysis was then repeated using each simulated level of Local clinician diagnosis misclassification (SX3 to SX20). The subgroup analysis was also repeated for each simulated level of Local clinician diagnosis misclassification for both the subgroup effect observed in the ENOS dataset, and for the increased subgroup effect created using simulation. Regression model coefficients and standard errors are presented on the log scale for ease of comparison.

## 3 Results

**Fig. 1:**
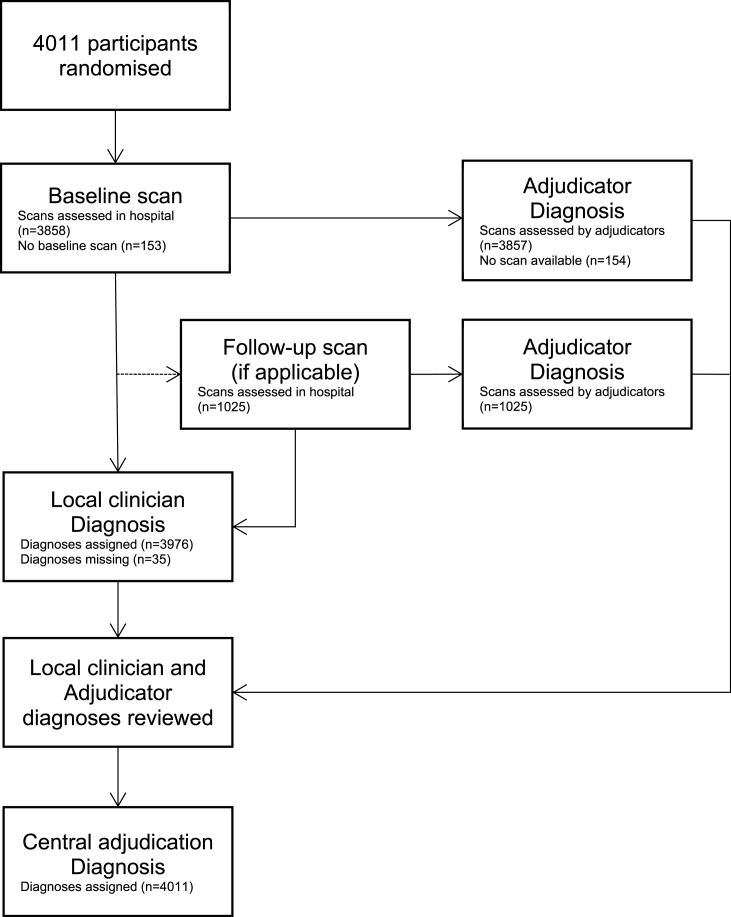
Flow diagram showing diagnosis of stroke type in ENOS.

Of 4011 participants randomised, 3857 (96%) and 1025 (26%) had baseline and follow up scans respectively that were assessed by adjudicators. A total of 35 participants had a missing Local clinician diagnosis, and all participants had a Central adjudication diagnosis assigned after the combined information from the hospital and central adjudicators was reviewed (Fig. 1).

The proportion of participants with each stroke type was similar for those that did or did not have a follow-up scan, indicating no evidence of bias in the selection of participants for a follow up scan and therefore having more information with which to assign a diagnosis (*see* Supplementary File, S2).

Agreement was high in ENOS, with local clinicians and central adjudicators agreeing on 79% of diagnoses at baseline. There was excellent agreement between Local clinician and Central adjudication diagnoses (crude agreement 98%, unweighted kappa, κ = 0.92) for the 3976 (99%) participants who could be included in this analysis (Table 1).

**Table 1:**
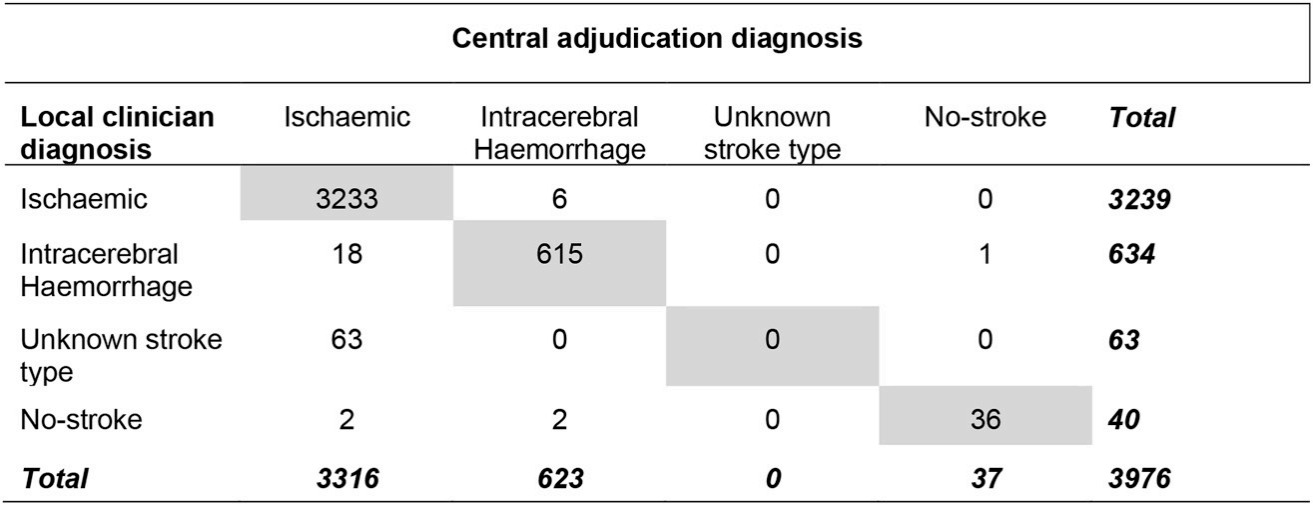
Agreement between Local clinician and Central adjudication diagnosis.

Crude agreement = 3884/3976 = 98%.

Unweighted kappa = 0.92.

Misclassification of Local clinician diagnosis resulted in kappa statistics for agreement between Central adjudication and Local clinician diagnoses of 0.78, 0.67, 0.46, 0.32 and 0.21 for SX3-SX20 respectively. As expected due to strong agreement between Central adjudication and Local clinician diagnoses of stroke type, it made little difference which one was used as a covariate in the primary analysis of observed ENOS data (p-value for homogeneity p = 0.95, *see* Supplementary File, S3). Similarly, coefficients and standard errors for the interaction between GTN and stroke type were very similar regardless of whether Local clinician or Central adjudication diagnosis of stroke type was used (*data not shown*).

Increased levels of non-differential misclassification of stroke diagnosis introduced by simulation made no material difference to the estimated treatment effect of GTN or the precision of the estimate (Table 2). Table 3 shows the effect of GTN separately for each stroke type using the magnitude of subgroup effect observed in the ENOS data, and where non-differential misclassification of stroke type is increased by simulation. The number of participants diagnosed with ischaemic stroke decreased, whilst each of the other types of stroke increased, respectively, with increasing misclassification. The effects of misclassification on stroke-specific estimates of GTN treatment were not wholly consistent, although increasing misclassification tended to give treatment effects closer to zero and standard errors that increased or decreased inversely with stroke-specific sample size accordingly.

**Table 2:** Effect of increased misclassification of stroke type at trial entry on ENOS primary analysis.

Source of diagnosis of stroke type at trial entry	Results from regression model comparing effect of GTN versus no GTN	
	Log OR	SE log OR
Central adjudication	−0.02473	0.05565
SX3	−0.02446	0.05563
SX5	−0.02426	0.05563
SX10	−0.02426	0.05561
SX15	−0.02411	0.05561
SX20	−0.02415	0.05561

SX3-SX20 refer to the misclassified Local clinical diagnoses. Kappa statistics showing the agreement between each diagnosis and Central adjudication diagnosis are 0.78, 0.67, 0.46, 0.32 and 0.21 for SX3-SX20 respectively.

**Table 3:** Effect of misclassification of stroke type at trial entry on subgroup analysis: based on subgroup effect observed in ENOS data.

Stroke Type	Source of diagnosis of stroke type at trial entry	N	Subgroup-specific estimated effect of GTN versus no GTN	
			Log OR	SE log OR
Ischaemic	Central adjudication	3338	−0.03048	0.06085
	SX3	3096	−0.03114	0.06003
	SX5	2935	−0.02953	0.06491
	SX10	2531	−0.02503	0.06987
	SX15	2129	−0.03130	0.07618
	SX20	1725	−0.03043	0.08476

Haemorrhagic	Central adjudication	623	0.02699	0.14110
	SX3	657	0.02761	0.13717
	SX5	682	0.01898	0.13474
	SX10	739	0.01443	0.12943
	SX15	798	−0.00496	0.12456
	SX20	855	0.00832	0.12027

Unknown	Central adjudication	1	–	
	SX3	196	0.00091	0.25320
	SX5	325	−0.01070	0.19592
	SX10	652	−0.03348	0.13830
	SX15	975	−0.00867	0.11286
	SX20	1302	−0.02353	0.09743

No-stroke	Central adjudication	38	0.18475	0.65491
	SX3	51	0.16043	0.54100
	SX5	58	0.05159	0.48825
	SX10	78	−0.00907	0.40673
	SX15	98	−0.00359	0.36080
	SX20	118	−0.00448	0.32877

Simulations produced datasets containing 4000 observations.

SX3-SX20 refer to the misclassified Local clinical diagnoses. Kappa statistics showing the agreement between each diagnosis and Central adjudication diagnosis are 0.78, 0.67, 0.46, 0.32 and 0.21 for SX3-SX20 respectively.

Simulation of a subgroup effect, whereby GTN was beneficial among participants with an ischaemic stroke, and harmful among participants with a haemorrhagic stroke, attenuated the treatment effects even further (Table 4). After stroke type was increasingly misclassified using simulation, statistical evidence of a subgroup effect was reduced and the effects of subgroup sample size on precision were as expected (Table 4, Table 5).

**Table 4:** Effect of misclassification of stroke type on subgroup analysis: based on simulated subgroup effect.

Stroke Type	Source of diagnosis of stroke type at trial entry	N	Subgroup-specific estimated effect of GTN versus no GTN	
			Log OR	SE log OR
Ischaemic	Central adjudication	3338	−0.14122	0.06085
	SX3	3096	−0.13885	0.06320
	SX5	2935	−0.13576	0.06493
	SX10	2531	−0.12591	0.06988
	SX15	2129	−0.11477	0.07624
	SX20	1725	−0.11388	0.08478

Haemorrhagic	Central adjudication	623	0.29183	0.14156
	SX3	657	0.25154	0.13760
	SX5	682	0.22485	0.13522
	SX10	739	0.17519	0.12990
	SX15	798	0.11796	0.12482
	SX20	855	0.08150	0.12015

Unknown	Central adjudication	1	–	
	SX3	196	−0.09800	0.25358
	SX5	325	−0.12723	0.19472
	SX10	652	−0.15382	0.13795
	SX15	975	−0.13565	0.11250
	SX20	1302	−0.12854	0.09744

No-stroke	Central adjudication	38	0.25680	0.68781
	SX3	51	0.14555	0.53944
	SX5	58	0.05832	0.49047
	SX10	78	0.07533	0.40879
	SX15	98	−0.05162	0.36561
	SX20	118	0.02436	0.33095

Simulations produced datasets containing 4000 observations.

SX3-SX20 refer to the misclassified Local clinical diagnoses. Kappa statistics showing the agreement between each diagnosis and Central adjudication diagnosis are 0.78, 0.67, 0.46, 0.32 and 0.21 for SX3-SX20 respectively.

**Table 5:** P-values for interaction tests between GTN and stroke type based on observed and simulated ENOS data.

Data source	Source of diagnosis	Median p-value from 100 simulated analyses (IQR)
*Subgroup effect based on observed ENOS data*	Central adjudication	0.38592 (0.17160, 0.61673)
	SX3	0.39858 (0.15347, 0.65161)
	SX5	0.46350 (0.16563, 0.72459)
	SX10	0.43609 (0.22501, 0.78923)
	SX15	0.54638 (0.32173, 0.79183)
	SX20	0.46829 (0.23353, 0.70323)
*Subgroup effect based on simulated ENOS data*	Central adjudication	0.00882 (0.00096, 0.06394)
	SX3	0.02801 (0.00699, 0.14882)
	SX5	0.04675 (0.00677, 0.24457)
	SX10	0.10912 (0.01997, 0.31892)
	SX15	0.16117 (0.05030, 0.47707)
	SX20	0.24764 (0.06521, 0.54910)

SX3-SX20 refer to the misclassified Local clinical diagnoses. Kappa statistics showing the agreement between each diagnosis and Central adjudication diagnosis are 0.78, 0.67, 0.46, 0.32 and 0.21 for SX3-SX20 respectively.

## 4 Discussion

Misclassification of stroke type by local trial site clinicians was low, with excellent agreement found between the Central adjudication and Local clinician diagnosis. Due to the level of agreement, there was little impact of adjudication of stroke type at trial entry on the primary analysis or subgroup analysis of ENOS. Increased levels of non-differential misclassification produced little change in the primary outcome. After simulating a strong subgroup effect by stroke type, increased misclassification resulted in reduction of the subgroup effect, suggesting that in this situation adjudication may be important to ensure robust results.

In ENOS, due to blinding, differential misclassification of stroke type was unlikely, which was why we introduced non-differential misclassification using simulation. Even with non-differential misclassification increased by 20 times the observed level, there was little effect on both the primary and subgroup analyses. Only when a substantial subgroup effect (p < 0.01) and marked misclassification of stroke diagnosis by local investigators were simulated would adjudication have resulted in differing conclusions. These extreme, and thus arguably unlikely, conditions before central adjudication is seen to add value are likely due to the fact that in our analyses, diagnosis of stroke type is a baseline variable rather than a study endpoint. However a recent Cochrane review [20] that assessed endpoint adjudication of subjective binary events across a range of clinical areas, including 47 RCTs, also found that adjudication did not affect the treatment effect estimates (Ratio of Odds Ratios: 1.00, 95% C.I: [0.97 to 1.04]). The review suggested that adjudication ‘may be most important when onsite assessors are not blinded and the risk of misclassification is high’.

It is worth noting that in ENOS, diagnostic adjudication was used for purposes in addition to informing the diagnosis. The adjudication process provided a large amount of extra information which hospital scan results would not have recorded. This information can be used to carry out imaging-based subgroup analyses or help to improve any subsequent sub-studies. Furthermore, the central adjudication process meant that each scan had been rated using a central, standard approach, enabling data to be pooled with other trials that have used a similar method. Therefore, the ENOS data can be utilised further, alongside existing data, to provide a larger sample size to test the independent prognostic value and potential treatment implications of the scan signs raised in various studies, as well as assisting in confirming or refuting ideas about not treating certain types of infarct or effects on infarct swelling.

The diagnostic adjudication process in ENOS resulted in increased complexity, and monetary and time costs. These included payments to adjudicators, resources associated with handling adjudicator data (data entry, database programming, and statistical analysis), the time taken by the trial team to determine the trial diagnosis, and data queries. Although this is the first study we are aware of to investigate diagnostic adjudication in stroke trials, where diagnosis is not used as an endpoint, previous studies which have looked at adjudication of endpoints have found similar conclusions. Slight benefits of improving accuracy and reducing misclassification were outweighed by the cost and complications introduced by an adjudication committee [2,11]. However, there may be some unmeasurable benefits of an adjudication process, and adjudication could have indirectly strengthened local assessment due to a policing effect. This effect could have resulted in improved site performance as investigators would have been aware that diagnoses would have been checked centrally, and thus perform more carefully.

One strength of this study is that we used a large, well conducted, randomised trial to provide data from over 4000 participants for analysis. Furthermore, the data completeness was extremely high, minimising the risk of bias due to partially completed data. The simulation undertaken in this study allowed an investigation into the robustness of observed results to more extreme data scenarios. This was important to understand how adjudication of diagnosis at trial entry could affect a similar trial where agreement was not as good as observed in ENOS. This approach, using a combination of observed and simulated data, can be readily applied to secondary analyses of other trials, notably on outcome variables as well as baseline variables, in order to inform future studies.

A limitation of this study is that the potential for adjudication to have an important effect is likely to be less for a baseline variable, as seen in ENOS, rather than a primary outcome as in Ninomiya et al. [11]. Therefore, we also looked into the impact of adjudication on subgroup analyses involving stroke type, to allow a thorough investigation into the value of central adjudication of a baseline variable had on ENOS. Furthermore, the treatment estimates for GTN for both ischaemic and haemorrhagic stroke were similar, so increased misclassification in this situation had limited impact, although this may not be the case in other studies where there is a treatment-diagnosis interaction. Simulation allowed us to explore this setting, but a further investigation using data from another large trial would be beneficial to reinforce our findings.

## 5 Conclusions

This study found that clinicians at ENOS trial sites largely were correct in their diagnosis of stroke and adjudication did not impact on the trial results. Adjudication of stroke type at trial entry would have altered conclusions had there been strong evidence of a subgroup effect by stroke type, and where misclassification was at least ten times that observed in ENOS. In pilot or feasibility studies, misclassification could be estimated in order to inform whether adjudication would be useful in that particular trial. Researchers should consider the value adjudication could bring to their study before its implementation in a clinical trial to avoid wasted time and unneeded expenditure.

## List of abbreviations

ENOS – Efficacy of Nitric Oxide in Stroke.

GTN – Glyceryl trinitrate.

## Ethics approval and consent to participate

Ethical approval was not required for this research due to it being a secondary analysis of the ENOS trial where ethical approval was attained. Written informed consent for the ENOS trial was obtained from each patient, or, in the case when the patient did not have capacity, from a relative or independent physician.

## Availability of data and materials

The datasets used and/or analysed during the current study are available from the corresponding author on reasonable request.

## Conflicts of interest

The authors declare that they have no competing interests.

## Role of the funding source

PJG is funded by a National Institute for Health Research Research Methods Fellowship (RMFI-2014-05-13). PMB is Stroke Association Professor of Stroke Medicine, and is a NIHR Senior Investigator. This paper presents independent research funded by the NIHR through PMB's Senior Investigator award. The views expressed are those of the authors and not necessarily those of the NHS, the NIHR or the Department of Health. The ENOS trial was primarily funded by UK Medical Research Council. The funding sources had no involvement in the study design, collection, analysis and interpretation of data.

## Appendix A Supplementary data

File S1 File S1

Data Profile Data Profile
