# Supplementary material for: Central masked adjudication of stroke diagnosis at trial entry offered no advantage over diagnosis by local clinicians: Secondary analysis and simulation
Source: Contemp Clin Trials Commun. 2018 Nov 10;12:176–81. doi: 10.1016/j.conctc.2018.11.002 (PMC6249966; doi:10.1016/j.conctc.2018.11.002)
Supplement: File S3 [file mmc1.docx]

| **Source of diagnosis of stroke type at trial entry** | **Results from regression model comparing effect of GTN versus no GTN** | | |
| --- | --- | --- | --- |
|  | **Odds Ratio** | **95% C.I** | **p-value** |
| Central adjudication | 1.01 | [0.91 to 1.13] | 0.81 |
| Local clinician | 1.01 | [0.90 to 1.13] | 0.86 |

**S3:**

Supplementary Table 2: Effect of increased misclassification of stroke type at trial entry on ENOS primary analysis

Test of homogeneity between estimates, p=0.95
