# Supplementary material for: Central masked adjudication of stroke diagnosis at trial entry offered no advantage over diagnosis by local clinicians: Secondary analysis and simulation
Source: Contemp Clin Trials Commun. 2018 Nov 10;12:176–81. doi: 10.1016/j.conctc.2018.11.002 (PMC6249966; doi:10.1016/j.conctc.2018.11.002)
Supplement: File S2 [file mmc2.docx]

|  | **Adjudicator diagnosis based on Baseline Scan (n=3857)** | | **Adjudicator diagnosis based on Follow-up Scans (n=1025)** |
| --- | --- | --- | --- |
|  | **No follow-up scan available (n=2832)** | **Follow-up scan available (n=1025)** |  |
| Ischaemic | 1516 (54%) | 556 (54%) | 644 (63%) |
| Intracerebral Haemorrhage | 403 (14%) | 184 (18%) | 182 (18%) |
| Ischaemic with Haemorrhagic transformation | 71 (3%) | 14 (2%) | 78 (8%) |
| Scan not normal, no acute lesion or non-stroke lesion | 759 (27%) | 253 (25%) | 113 (11%) |
| No-stroke | 7 (<1%) | - | 2 (<1%) |
| Normal scan | 76 (3%) | 18 (2%) | 6 (1%) |

**S2:**

Supplementary Table 1: Adjudicator diagnosis of stroke type at trial entry based on baseline and follow-up scans
