# Supplementary material for: Central masked adjudication of stroke diagnosis at trial entry offered no advantage over diagnosis by local clinicians: Secondary analysis and simulation
Source: Contemp Clin Trials Commun. 2018 Nov 10;12:176–81. doi: 10.1016/j.conctc.2018.11.002 (PMC6249966; doi:10.1016/j.conctc.2018.11.002)
Supplement: File S1 [file mmc3.docx]

S1 - Simulation methods

**Aims**

The objective of the simulation study was to investigate the extent of misclassification of Local clinician diagnosis needed before adjudication changed trial conclusions in ENOS or a similar large acute stroke trial. Therefore, statistical simulations were created to:

1. Increase the extent of misclassification of Local clinician diagnosis of stroke compared with the gold standard Central adjudication diagnosis;
2. Introduce an interaction (subgroup effect) between ENOS treatment arm and stroke type

These simulations enabled us to investigate the effects of misclassification on the ENOS primary analysis and on subgroup analysis, for both the subgroup effect observed in ENOS and for a subgroup effect introduced by simulation. The magnitude of the treatment-stroke type interaction was increased in simulation as there was no statistical evidence of a subgroup effect in the observed ENOS dataset.

**Data generating mechanisms**

A simulated dataset that closely replicated the ENOS data was created through random sampling of a multinomial distribution. This abridged dataset contained only key variables that allowed investigation of the importance of adjudication for the primary analysis (functional outcome after 90 days measured on mRS, treatment group) and for the subgroup analysis by stroke subtype (Local clinician diagnosis, Central adjudication diagnosis). This simulated dataset maintained the relationship between all variables and was based on the real ENOS data. The estimated coefficient and standard error for the treatment effect of GTN in this simplified dataset was only marginally different from that estimated from the full ENOS dataset.

**Misclassification of Local clinician diagnosis**

Misclassification of Local clinician diagnosis was increased as follows. The probability, $p_{ij}$, of a participant being incorrectly classified as stroke type $i$ for their Local clinician diagnosis, given they had a specific stroke type $j$ diagnosed for their Central adjudication diagnosis, was determined ($i,j=1,\ldots, 4, i\neq j)$. These probabilities were multiplied by a positive scale factor $x$, and a new diagnosis was simulated which mimicked the Central adjudication diagnosis, but which had $\left( \sum_{\begin{aligned} i=1 \\ i\neq j \end{aligned}}^{4} \sum_{j=1}^{4} p_{ij} \right)x$ participants misclassified. This new diagnosis had the same pattern of misdiagnosis as Local clinician diagnosis, but had $x$ times the level of misclassification observed in ENOS.

**Introducing subgroup effect by stroke type**

A subgroup effect between treatment and stroke type was introduced by reducing mRS score by 1 point for 10% of participants with an Ischaemic stroke, and increasing mRS score by 1 point for 30% of participants with an Intracerebral Haemorrhage. mRS scores for all participants were constrained to be in the normal range of 0 to 6. All participants with an altered mRS score were in the GTN arm of the trial.

**Methods**

**Number of simulations needed**

To determine the amount of simulations to run to produce a pre-specified level of accuracy, a formula provided by Burton et al.^[1]^ can be used:

$$N=\left( \frac{Z_{1-(\frac{\alpha}{2})}\sigma}{\delta} \right)^{2}$$

Here, $N$ is the number of simulations required, $Z_{1-\left( \frac{\alpha}{2} \right)}$ is the 1 – (α/2) quartile of the standard normal distribution, σ^2^ is the variance of the main parameter of interest and δ is a specified level of accuracy for the simulation to be within for the main parameter of interest, measured in %. In our study, the main parameter of interest was the estimated treatment effect of GTN fitted in an ordinal logistic regression model, with Central adjudication diagnosis as the only covariate. We estimated σ from the observed trial data, a significance level of 5% was assumed and a 1% level of accuracy was used. Thus, with σ = 0.05, α=0.05, and δ=0.01, the number of simulations needed,$N$, was 96, which we rounded up to 100.

**Amount of misclassification**

In simulated datasets, the misclassification of Local clinician diagnosis observed in ENOS was increased by factors of 3, 5, 10, 15 and 20 (referred to as SX3, SX5, SX10, SX15 and SX20 respectively). This resulted in kappa statistics for agreement between Central adjudication and Local clinician diagnoses of 0.78, 0.67, 0.46, 0.32 and 0.21 for SX3-SX20 respectively.

**Undertaking simulations and combining estimates**

For each simulated dataset, five new diagnoses (SX3-SX20) and one new outcome (mRS with introduced stroke type interaction) were generated. Following this, the primary analysis was undertaken using each diagnosis, and the subgroup analysis by stroke type was computed for each diagnosis and for the observed ENOS interaction as well as the simulated stroke type interaction. Therefore, there were 18 analyses undertaken on each simulated dataset.

To combine the estimates from the 100 simulated datasets into a single representative estimate, the mean of the treatment effect and mean of the estimated within simulation standard error were used. The median and interquartile range were used to describe the p-values from each test of interaction, due to their skewed distributions.

**References**

[1] Burton A, Altman D, Royston P, Holder RL. The design of simulation studies in medical statistics. Statistics in Medicine 2006;25:4279-4292.
